# Electrolyte supplementation for gestating beef cows under heat stress conditions: cow-calf performance, thermotolerance and physiological responses

**DOI:** 10.1093/tas/txag031

**Published:** 2026-03-16

**Authors:** Isabelle P Siqueira, Marcelo Vedovatto, Juliana Ranches, Giancarlo P Silva, Barbara R dos Reis, Hiam Marcon, Ashley K Edwards, Nate Haas, Matheus L Ferreira

**Affiliations:** Hill Farm Research Station, Louisiana State University, Homer, LA, 71040, United States; Dean Lee Research and Extension Center, Louisiana State University, Alexandria, LA, 71302, United States; Eastern Oregon Agricultural Research Center, Oregon State University, Burns, OR, 97720, United States; Hill Farm Research Station, Louisiana State University, Homer, LA, 71040, United States; White Sand Research Unit, Mississippi State University, Poplarville, MS, 39470, United States; Dean Lee Research and Extension Center, Louisiana State University, Alexandria, LA, 71302, United States; Department of Animal Science, Sao Paulo State University, Jaboticabal, SP, 14884-900, Brazil; Dean Lee Research and Extension Center, Louisiana State University, Alexandria, LA, 71302, United States; Bio-Vet, Inc. Barneveld, WI, 53507, United States; Hill Farm Research Station, Louisiana State University, Homer, LA, 71040, United States

**Keywords:** acid-base balance, grazing, hydration, metabolism, water intake

## Abstract

Electrolyte supplementation has been proposed as a strategy to mitigate heat stress by supporting acid–base balance and hydration; however, its application in pasture-based beef cow–calf systems has not been evaluated. This study assessed the effects of providing an electrolyte-fortified water solution to late-gestation beef cows managed on pasture during prolonged summer heat stress. Fifty-four fall-calving Angus-cross cows [(**BW**) = 582 ± 8.43 kg] and body condition score [(**BCS**) = 5.9 ± 0.1] were assigned to 10 bermudagrass pastures and allocated to: (1) Control (water only) or (2) Electrolyte-supplemented water (Bovine GoldLyte; 25 g/cow/day; *n* = 5 pastures/treatment) for approximately 90 days prepartum. Cow BW, BCS were collected on d 0, 28, 56, 84 and 168, and blood on d 0, 14, 28, 56 and 84. Water and electrolyte intake were collected three time a week from day 0 to 84. Respiration rate was measured every 14-d and intravaginal temperatures were measured from d 28 to 35 and d 56 to 63. Electrolyte intake averaged 26.7 ± 1.21 g/cow/day. Supplementation did not affect cow BW, BCS, calf birth weight, respiration rate, intravaginal temperature, or water intake (*P* ≥ 0.51). Control cows consumed more mineral-mix from weeks 5 to 10 (treatment × day; *P* < 0.001). Electrolyte supplementation did not alter blood pH, pCO_2_, Na^+^, K^+^, ionized Ca, or hemoglobin (*P* ≥ 0.16), but cows receiving electrolytes showed decreased hematocrit (*P* = 0.09) and increased bicarbonate (*P* = 0.06), base excess (*P* = 0.04), and tCO_2_ (*P* = 0.04). Cows supplemented with electrolyte had decreased albumin concentrations (treatment × day, *P* = 0.03) on day 56 (*P* = 0.09) and 84 (*P* = 0.01). In conclusion, electrolyte supplementation enhanced hydration and buffering capacity in heat-stressed gestating beef cows, but these physiological changes did not translate into improved performance or thermotolerance. Reduced mineral intake among supplemented cows may have offset potential benefits, suggesting that continuous electrolyte supplementation offers limited practical value for improving productivity or heat resilience in pasture-based beef systems.

## Teaser

Electrolyte supplementation for beef cows during the summer improved hydration and blood buffering capacity but did not improve performance or thermotolerance.

## Introduction

Heat stress is an increasing concern for livestock production, as rising temperatures are expected throughout the twenty-first century (Thornton et al. 2022). In cattle, heat stress reduces productivity, health, and reproductive efficiency (West, 2003; Collier et al. 2006), leading to substantial economic losses (St-Pierre et al. 2003). Physiologically, heat stress triggers responses such as elevated body temperature and respiration rate (West, 2003; Ferreira et al. 2025), often accompanied by increased sweating and evaporative cooling. These processes result in significant water and electrolyte losses (Cardoso et al. 2015), which disrupt homeostasis, increase maintenance requirements, and contribute to acid-base imbalances (West et al. 1991; Al-Qaisi et al. 2020).

Electrolyte supplementation has the potential to mitigate metabolic effects of heat stress by improving hydration, supporting acid-base balance, and enhancing thermotolerance (West et al. 1992; Al-Qaisi et al. 2020). Previous research has evaluated electrolyte use in cattle subjected to prolonged stress after transportation (Schaefer et al. 1990; Zhao et al. 2022) and in heat-stressed dairy cows (Schneider et al. 1984; Mallonée et al. 1985; Wildman et al. 2007). Increasing buffering capacity with electrolytes has been linked with improvements in dry matter intake and milk yield (West et al. 1992) and blood acid-base balance (Al-Qaisi et al. 2020) in heat-stressed dairy cows.

While nutritional and management strategies to cope with heat stress has been extensively studied in dairy production (Collier et al. 2006; Oliveira et al. 2025), research on beef cow-calf systems remain limited (Ferreira et al. 2025), and effective mitigation strategies are scarce. Dairy cows are typically managed in housing systems where cooling technologies and nutritional interventions can be implemented (Oliveira et al. 2025). In contrast, pasture-based systems offer fewer nutritional and management options, relying primarily on natural or artificial shade, which may be insufficient during severe summer conditions (Edwards-Callaway et al. 2021).

Despite numerous studies investigating the use of electrolyte supplementation to mitigate the physiological effects of heat stress (Schneider et al. 1984; Mallonée et al. 1985; Al-Qaisi et al. 2020), none have addressed its application or effects in grazing beef cow-calf systems. Consequently, the potential benefits and outcomes of using this strategy in such systems remain unexplored. We hypothesized that the supplementation with electrolyte solution to gestating beef cows managed on pasture during prolonged heat stress conditions would improve acid-base balance, replenish electrolytes, and improve hydration status. Therefore, the objective of this study was to evaluate the effects of electrolyte supplementation during late gestation under heat stress conditions on cow performance, thermotolerance, and physiological responses.

## Material and methods

The experiment was conducted at the Hill Farm Research Station, Louisiana State University Agricultural Center, Homer, Louisiana (32°45′18″N to 93°04′25″W) from June 2025 to December 2025. All animal handling and procedures were approved by the Institutional Animal Care & Use Committee from Louisiana State University (protocol A2025-05).

### Animals and experimental design

Fifty-four fall-calving Angus-cross cows (6 ± 2 yrs of age, ∼ ⅞ Angus and ⅛ Brangus) were stratified by BW (582 ± 8.43 kg) and BCS (5.9 ± 0.1) and allocated into 1 to 10 bermudagrass pastures ‘common and coastal’ (*Cynodon dactylon*; 3 to 5 ha pastures/5 or 6 cows per pasture) at approximately 90 days pre-partum (d 0). Cows used in the present study were confirmed pregnant by natural service in the previous breeding season with bulls rotating every 28 d.

Treatments were randomly assigned to pastures as following: **(1)** Control: access to water only, or **(2)** Electrolyte supplementation: water fortified with an electrolyte solution (Bovine GoldLyte, Bio-Vet, Barneveld, WI), from day 0 to 90 [average day of calving (90 ± 10 d prepartum)]. The electrolyte supplement contained: 12.3% salt, 8.5% Na, 0.44% Mg, 26.5% K, 300 mg/kg Cu, 1400 mg/kg Zn, 1400 mg/kg Fe, 1400 mg/kg Mn, and 40 mg/kg Co, 3,500,000 IU vitamin A, 700,000 IU vitamin D_3_, 5000 IU vitamin E, 1500 mg niacin, and 3000 mg choline. Electrolyte solution was diluted directly into the water tanks at a ratio of 25 g per total daily water intake per cow, as per manufacturer’s recommendation.

Cows were moved to their assigned pastures 14 days before study initiation for an adaptation period and baseline water intake monitoring to calculate the electrolyte solution preparations. Water consumption was measured using electronic turbine flow meters (Generic, WG-01, Guangdong, China) installed on each pasture’s water-tank inlet line (Williams et al. 2020) upstream of the float valve, allowing the volume of water entering the trough to be measured. Water tanks (Rubbermaid, Atlanta, GA; *n* = 6 and CountyLine, Brentwood, TN; *n* = 4) were placed over a concrete platform (2 m x 3 m) to ensure proper float-valve function. Each flow meter was calibrated before installation and tested, showing an accuracy of 98.9% for water measurement (Supplementary File 1). Accuracy testing involved 20 measurements in which water was removed from the tank, its volume manually measured and then compared to the corresponding readings from the flow meter.

A complete free choice mineral mix was provided to all pastures in mineral troughs (14.8% Ca, 7.5% P, 18.8% NaCl, 1% Mg, 1% K, 3600 mg/kg Zn, 3600 mg/kg Mn, 1200 mg/kg Cu, 12 mg/kg Co, 60 mg/kg I, 27 mg/kg Se, 662 IU/g vitamin A, 66 IU/g vitamin D and 0.66 IU/g vitamin E; Purina Wind & Rain Storm All-Season 7.5 Complete) with weekly allowances restricted based on a targeted intake of 100 g per cow per day. Mineral mix offered and refusals were weighed weekly, dried in a forced-air oven at 55°C for 72 h, to estimate mineral intake per pasture.

All cows had access to artificial shade which provided on average approximately 4.9 m^2^ of shade per animal/pasture, which is greater than the recommendation of shade for cows (Armstrong, 1994). At calving (∼ d 90), birth date, birth weight and calf sex were recorded. After calving, all cows were moved to Bermuda pastures with natural shade, offered free-choice access to Bermudagrass hay and managed as a single group until the breeding season (d 168).

## Data collection

### Water and electrolyte solution intake

Water and electrolyte solution intake were monitored three time a week by recording the disappearance of each fluid (water and electrolyte solution) over a 24-hour period, as previously described during the adaptation period using the flow meters. To prevent unintended dilution of the electrolyte solution throughout the day as cows consumed water, the inlet valve of each water tank was closed and reopened twice at 1100 h and 1700 h on measurement days. Following measurements, fresh water and a newly prepared electrolyte solution were provided to each trough. The electrolyte-to-water dilution ratio was adjusted at every measurement based on the flow information. Total daily intake was calculated as the sum of morning and afternoon water or electrolyte solution consumption. On days when direct water intake measurements were not obtained, the electrolyte inclusion rate was calculated based on the most recent intake data available. To maintain as uniform an electrolyte concentration as possible across days and reduce dilution effect in non-measurement days, electrolyte was also mixed and delivered twice daily always at 1100 h and 1700 h using previous flow information.

Two identical water tanks to those used in the pastures were placed in a representative area of the experimental area and filled with water and electrolyte solution to estimate evaporation losses. Evaporation losses were measured in a span of a week, and averaged to estimate water loss per day, which was then used to adjust water and electrolyte solution intake calculations.

### Cow performance, metabolism and thermotolerance

Cow full BW and BCS (1–9) were collected on d 0, 28, 56, 84 (near calving) and 168 (start of breeding season). Cow blood samples were collected on d 0, 14, 28, 56, 84 via jugular vein using commercial heparinized vacuum tubes for plasma harvest and tubes containing clot activator for serum harvest (BD Vacutainer, 10 mL; Becton, Dickinson and Company, Franklin Lakes, NJ). Blood samples were placed on ice immediately following collection. Tubes for serum harvest were centrifuged at 2500 × g for 30 min at 4°C and frozen at -20°C and stored until further analysis. Serum samples were analyzed for concentrations of albumin, total proteins, globulins, urea, glucose and creatinine. Heparinized whole blood was stored at 4°C and analyzed 30 min after collection for pH, partial pressure of carbon dioxide (**pCO_2_**), partial pressure of oxygen (**pO_2_**), sodium (**Na^+^**), potassium (**K^+^**), ionized calcium (**iCa**), bicarbonate (**${\text{HCO}}_{3}^{-}$**), base excess (**BE**), total concentration of CO_2_ (**tCO_2_**), hematocrit, and hemoglobin.

Respiration rate (**RR**) was measured individually in each cow at the pastures every 14 days until d 84 by visually counting flank movements of each cow for 1 minute (Brown-Brandl et al. 2005). Intravaginal temperatures were collected from cows randomly selected on day 0 (15 cows per treatment, 3 cows per pasture). Data was collected every 30 min from d 28 to d 35 and d 56 to d 63 (7 days consecutive in each sampling time), using implantable loggers (iButton DS1921H-F5#; resolution of ± 0.5°C; iButtonLink Technology, Whitewater, MI). Implantable loggers were placed intravaginally with a blank (hormone-free) controlled internal drug release device (Easy-Breed CIDR, Zoetis, Kalamazoo, MI). Intravaginal temperature data collected on the days of sensor insertion and removal were excluded from the statistical analysis to avoid confounding effects associated with handling and movement to and from pastures. The remaining intravaginal temperature data were averaged in 30-minute intervals and then further averaged across d 29 to 34 and d 55 to 62 prior to statistical analysis.

### Environmental data

Pastures were sampled to determine herbage mass, herbage allowance and forage nutritive value on d 0, 28, 56 and 84. Hand-plucked samples were used to evaluate forage nutritive value. Herbage mass (kg DM/ha) and herbage allowance (kg DM/kg BW) were calculated using the double sampling technique (Gonzalez et al. 1990).

Ambient temperature and relative humidity were measured using a U23-001A data logger (Onset Computer Corp., Bourne, MA) every hour during the entire study. Precipitation was daily measured using a standard rain gauge. Temperature and humidity parameters were used to calculate the thermal-humidity index (**THI**) and estimate potential heat stress.

THI data was calculated daily according to (Mader et al. 2006):


THI=(0.8 ×T)+(RH ÷100)×(T-14.4)+46.4,

where: T = ambient temperature (C°), RH = relative humidity (%).

**Figure 1:**
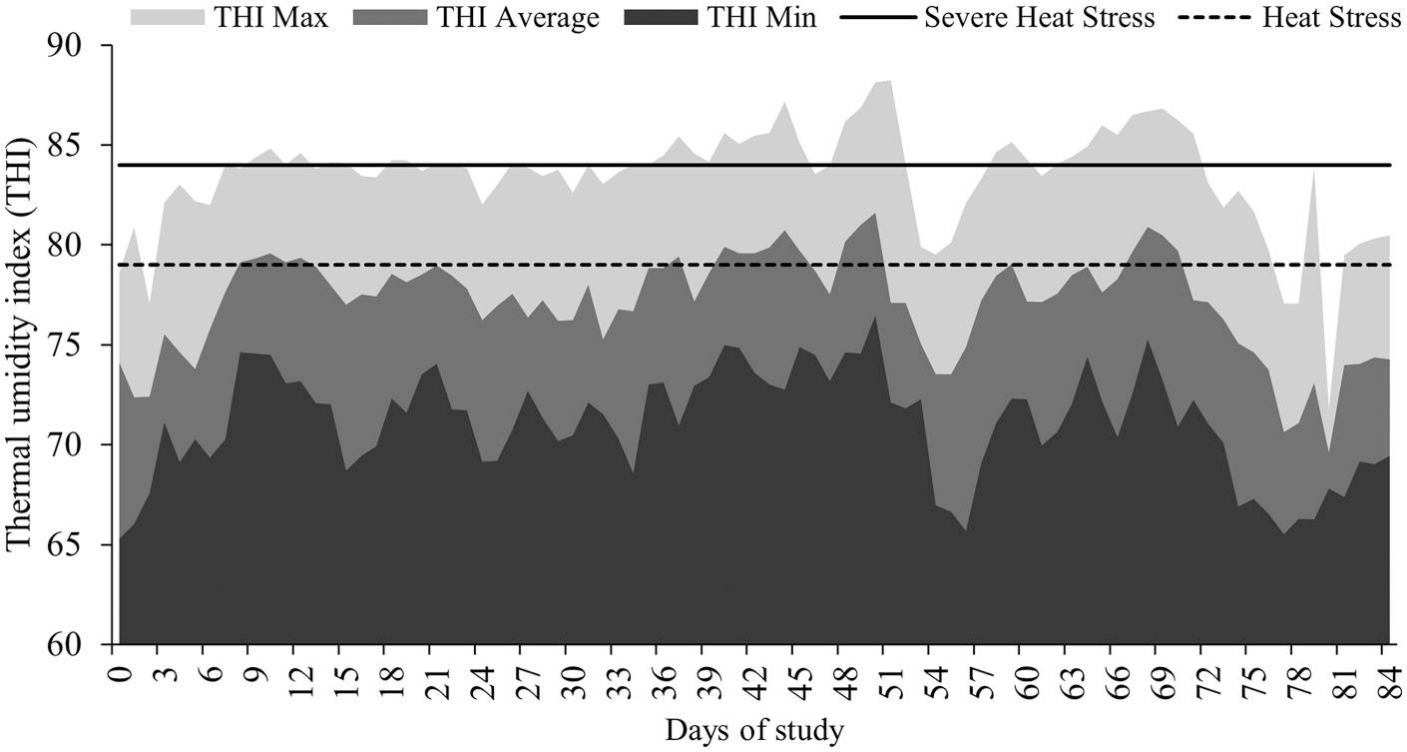
Maximum, average and minimum daily THI from days 0 to 84 of the study. Calculated according to Mader et al. (2006) as: THI = (0.8 × T) + (%RH ÷ 100) × (T—14.4) + 46.4. Major heat stress, 79 ≤ THI < 84; and severe, THI ≥ 84, according to (LCI, 1970). during pre-calving, 20 days were reported as major heat stress and 54 reported as moderate based on average THI.

The THI values were interpreted according to the Livestock Weather Safety Index (LCI, 1970) for heat stress potential as: minimal, ≤ 74; moderate, 74 < THI < 79; major, 79 ≤ THI < 84; and critical, THI ≥ 84. Average, minimum, and maximum THI observed were averaged and reported daily (Fig. 1). During pre-calving, 20 days were reported as major heat stress and 54 reported as moderate based on average THI.

### Laboratory analysis

Blood pH, pCO_2_, pO_2_, Na^+^, K^+^, iCa, hematocrit, and hemoglobin were determined using an i-STAT blood analyzer (Zoetis, Parsippany, NJ) with CG8 + cartridges. Bicarbonate was calculated according to Siggaard-Andersen (1963): ${\text{HCO}}_{3}^{-}$ [mmol/L] = 0.0307 × pCO2 × 10^(pH−6.129)^. Base excess was calculated as BE [mmol/L] = 0.93 × [14.83 × (pH—7.40) – 24.4 + ${\text{HCO}}_{3}^{–}$], according to NCCLS (1994). Total concentration of CO_2_ (tCO_2_) was calculated as the sum of dissolved CO_2_ and bicarbonate, as following: tCO_2_ [mmol/L] = ${\text{HCO}}_{3}^{–}$ + (0.0307 × pCO_2_). The i‑STAT system conducts automatic internal quality control for each cartridge, including sensor calibration verification, fluidics checks, and electronic function tests. According to manufacturer specifications, the i‑STAT system demonstrates analytical CVs typically < 5–7% for these analytes.

Serum samples were analyzed for concentrations of albumin, total protein, urea, glucose and creatinine concentrations using Beckman Coulter colorimetric and enzymatic colorimetric reaction kits in a Beckman Coulter AU480 Chemistry Analyzer (Beckman Coulter Inc., Brea, CA). Globulins were determined by subtracting albumin from total protein. Serum urea N (SUN) was calculated as 46.67% of blood urea. Internal quality control and verification of performance for each assay were performed and inter-assay coefficients of variation were consistently < 10% for all analytes.

### Statistical analysis

Data was analyzed using the MIXED procedure of SAS (SAS Institute Inc., Cary, NC, USA, version 9.4) with Kenward-Roger method of approximation to adjust degrees of freedom for the test of fixed effects. Pasture (treatment) was considered the experimental unit for all analysis. Cow and calf variables were analyzed for the fixed effects of treatment, day, and treatment × day interaction. Sampling day was considered repeated measures over time for cow BW, BCS, mineral intake, blood variables, herbage mass and allowance, and sampling hour for intravaginal temperatures. For repeated measures, cow was the subject for BW, BCS, blood variables and intravaginal temperature, and pasture for herbage mass, herbage allowance and mineral intake. The best covariance structure was chosen based on the lowest Akaike Information Criterion specific for each variable (chosen from compound symmetry, heterogeneous compound symmetry, autoregressive, and heterogeneous autoregressive). Cow BW, BCS and age on day 0 were used as covariates for these variables. Calf sex and calving day were included as a covariate in calf birth weight. Least square means were separated using least significant difference. Significance was set at *P* ≤ 0.05, and tendencies declared when *P* > 0.05 and ≤ 0.10.

## Results

Effects of day of the study (*P* < 0.02), but not treatment, or treatment × day (*P* ≥ 0.14). was detected for herbage mass, herbage allowance, and most forage chemical composition (Table 1). Herbage mass was greatest on d 84, followed by intermediate values on d 28 and 56, and was lowest on d 0 (*P* < 0.01; Table 1). Herbage allowance peaked on d 56, with intermediate values on d 28 and 84, and the lowest values on d 0 (*P* < 0.0001; Table 1). Forage CP concentrations were highest on d 0, intermediate on d 28 and 56, and lowest on d 84 (*P* < 0.001; Table 1). NDF concentration increased on d 84 compared with all other days, was intermediate on d 56 and d 0, and was lowest on d 28 (*P* = 0.01; Table 1). No differences between days were observed for TDN (*P* = 0.52; Table 1). Forage NEm and NEg were lowest on d 84 (*P* ≤ 0.02; Table 1).

**Table 1:** Average chemical composition, herbage mass and allowance of bermudagrass pastures.

Items^1^		Day of the study				SEM	*P*-value
	Hay	0	28	56	84		Day
***CP, %***	8.8	14.3^a^	12.0^b^	12.2^b^	11.3^c^	0.37	<.0001
***NDF, %***	70.7	64.6^bc^	63.4^c^	65.7^b^	67.0^a^	0.72	0.01
***TDN, %***	54.0	59.5	59.5	59.0	59.0	0.23	0.52
***NEm, Mcal/kg of DM***	1.00	1.20^a^	1.21^a^	1.19^a^	1.17^b^	0.008	0.01
***NEg, Mcal/kg of DM***	0.45	0.63^b^	0.64^a^	0.62^b^	0.60^c^	0.007	0.02
***Herbage mass, kg DM/ha***	–	2326^c^	3429^b^	3430^b^	3715^a^	220	<.0001
***Herbage allowance, kg DM/kg BW***	–	4.83^c^	7.01^b^	8.09^a^	6.36^b^	0.50	<.0001

1 CP, crude protein; NDF, neutral detergent fiber; TDN, total digestible nutrients; NEm, net energy for maintenance calculated as described by Weiss et al. (1992); NEg, net energy for gain calculated using the equations proposed by the NASEM (2016). Herbage mass (HM) was determined using the double sampling technique described by Gonzalez et al. (1990). Herbage allowance (HA) was determined by dividing the total pasture body weight by the herbage mass (Sollenberger et al. 2005). Effect of day of the study (*P* < 0.02), but not treatment and treatment × day (*P* ≥ 0.14), were detected for herbage mass, herbage allowance, and nutrient composition.

**Figure 2:**
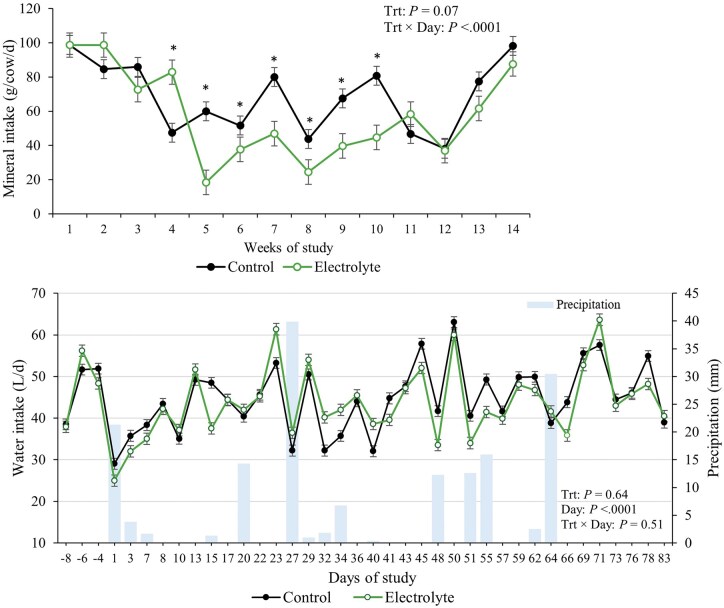
Mineral intake (Panel A) and water and electrolyte solution intake (Panel B) of beef cows supplemented or not supplemented with an electrolyte solution (5 pastures/treatment and 5 or 6 cows/pasture) from d 0 to 90 (90 ± 10 d pre-partum) under heat stress conditions. *Shows difference between treatments within days (*P* ≤ 0.05).

No effects of treatment, or treatment × day interaction was detected for cow BW and BCS (*P* ≥ 0.58; Table 2). Additionally, no effect of treatment was detected for calving day (*P* = 0.18) and calf birth weight (*P* = 0.88; Table 2). There was an interaction between treatment and day for mineral intake (*P* < 0.001), where control cows consumed more mineral in loose form than electrolyte-treated cows from wk 5 to wk 10 of the study and consumed less on wk 4 (Fig. 2a). Consumption of electrolyte was calculated as 26.7 (±1.21) g/cow/day. No effects of treatments treatment × day interaction were observed for water intake (*P* ≥ 0.56, Table 2 and Fig. 2b) and water intake per kg of BW (*P* ≥ 0.51).

**Table 2:** Performance, water, electrolyte and mineral intake of beef cows supplemented or not supplemented with an electrolyte solution (5 pastures/treatment and 5 or 6 cows/pasture) during gestation under heat stress conditions.

Item^1^	Treatments		SEM	*P*-value^2^	
	Control	Electrolyte		Trt	Trt × Day
***Body weight, kg***				0.61	0.68
*** d0***	580	584	10.02	–	–
*** d28***	608	593	10.02	–	–
*** d56***	632	617	10.02	–	–
*** d84***	653	653	10.02	–	–
*** d168***	601	615	10.02	–	–
***Body condition score, 1–9***				0.58	0.87
*** d0***	5.9	6.0	0.125	–	–
*** d28***	6.1	6.1	0.125	–	–
*** d56***	6.2	6.2	0.125	–	–
*** d84***	6.0	6.0	0.125	–	–
*** d168***	5.3	5.4	0.125	–	–
***Electrolyte intake, g/cow/d***	–	26.7	1.21	–	–
***Water intake, L/cow/d***	45.1	44.2	4.453	0.65	0.56
***Water intake, L/kg BW/d***	0.035	0.034	0.003	0.64	0.51
***Mineral intake, g/d/cow***	71.6	56.3	0.005	0.07	<0.001
***Calving day, day of study***	107	112	2.83	0.18	–
***Calf birth weight, kg***	33.5	33.8	1.07	0.88	–

1 Mineral offered and refusals were weighed weekly, dried in a forced-air oven at 55°C for 72 h, to estimate mineral intake per pasture and showed as intake per cow.

2 Sex (*P* = 0.01) and age/calving day (*P* = 0.20) were included as covariates for calf birth weight. day 0—start of study, day 84—near calving and d 168—start of the breeding season.

No effects of treatment, or treatment × day interaction (*P* ≥ 0.16) were detected for blood pH, pCO_2_, pO_2_, SO_2_, iCa, Na, K and hemoglobin (Table 3). Cows supplemented with electrolyte tended to have lower hematocrit (*P* = 0.09), tended to have greater blood concentrations of HCO_3_ (*P* = 0.06; Table 3), greater BE (*P* = 0.04) and tCO_2_ (*P* = 0.04; Table 3).

**Table 3:** Blood metabolites and acid-base balance of beef cows supplemented or not supplemented with an electrolyte solution (5 pastures/treatment and 5 or 6 cows/pasture) during gestation under heat stress conditions.

Item^1^	Treatments		SEM	*P*-value	
	Control	Electrolyte		Trt	Trt × Day
	*acid-base and hydration*				
***pH***	7.49	7.48	0.008	0.47	0.35
***pCO_2_, mmHg***	35.80	36.30	0.672	0.64	0.58
***pO_2_, mmHg***	34.46	34.91	1.003	0.75	0.16
***HCO_3_, mEq/L***	26.66	27.76	0.375	0.06	0.98
***BE, mEq/L***	3.32	4.56	0.434	0.04	0.95
***SO_2_, %***	70.31	70.70	1.561	0.86	0.16
***tCO_2_, mEq/L***	27.82	28.89	0.372	0.04	0.94
***iCa, mmol/L***	1.13	1.15	0.001	0.55	0.28
***Na, mEq/L***	143.30	143.10	0.369	0.59	0.23
***K, mEq/L***	3.70	3.80	0.047	0.64	0.79
***Hematocrit, %PCV***	34.12	32.20	0.713	0.09	0.28
***Hemoglobin, g/dL***	11.64	11.39	0.284	0.54	0.42
	*blood metabolites*				
***Glucose, mg/dL***	74.0	71.0	1.901	0.25	0.68
***Total proteins, g/dL***	8.00	7.95	0.163	0.75	0.77
***Albumin, g/dL***	3.43	3.41	0.028	0.89	0.03
***Globulins, g/dL***	4.56	4.52	0.126	0.76	0.25
***SUN, mg/dL***	7.62	7.31	0.227	0.32	0.27
***Creatinine, mg/dL***	1.41	1.46	0.044	0.44	0.13

1 pCO_2,_ Partial pressure of carbon dioxide; pO_2,_ Partial pressure of oxygen; HCO_3,_ Bicarbonate; BE, Base Excess; SO_2_, Oxygen saturation; tCO₂, Total carbon dioxide, iCa, Ionized calcium, Na, Sodium; K, Potassium; SUN, Serum Urea N.

**Figure 3:**
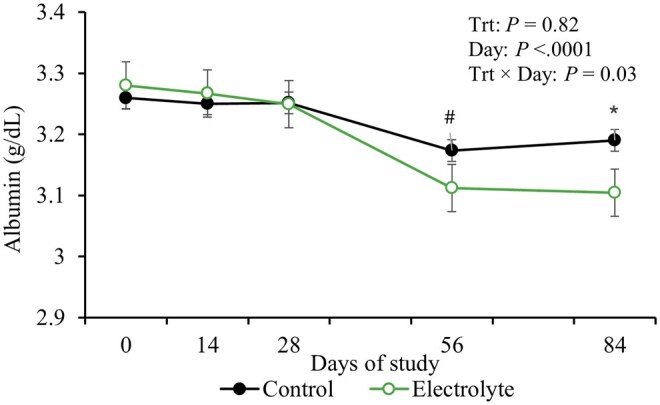
Blood albumin concentrations of beef cows supplemented or not supplemented with an electrolyte solution (5 pastures/treatment and 5 or 6 cows/pasture) from d 0 to 90 (90 ± 10 d pre-partum). Interaction between treatment and day were observed (*P* = 0.03), where albumin concentrations were decreased for electrolyte cow on days 56 (*P* = 0.09) and 84 (*P* = 0.01). *Shows difference between treatments withing day (*P* ≤ 0.05) and ^#^shows tendency (*P* > 0.05 and *P* ≤ 0.10).

No effects of treatments (*P* ≥ 0.25) or treatment × day interaction (*P* ≥ 0.13) were observed for glucose, SUN, total proteins, globulins and creatinine (Table 3). Interaction treatment and day was observed for albumin (*P* = 0.03; Table 3), where cows supplemented with electrolyte tended to have less albumin concentrations on day 56 (*P* = 0.09) and less on 84 (*P* < 0.001; Fig. 3) compared to control.

**Figure 4:**
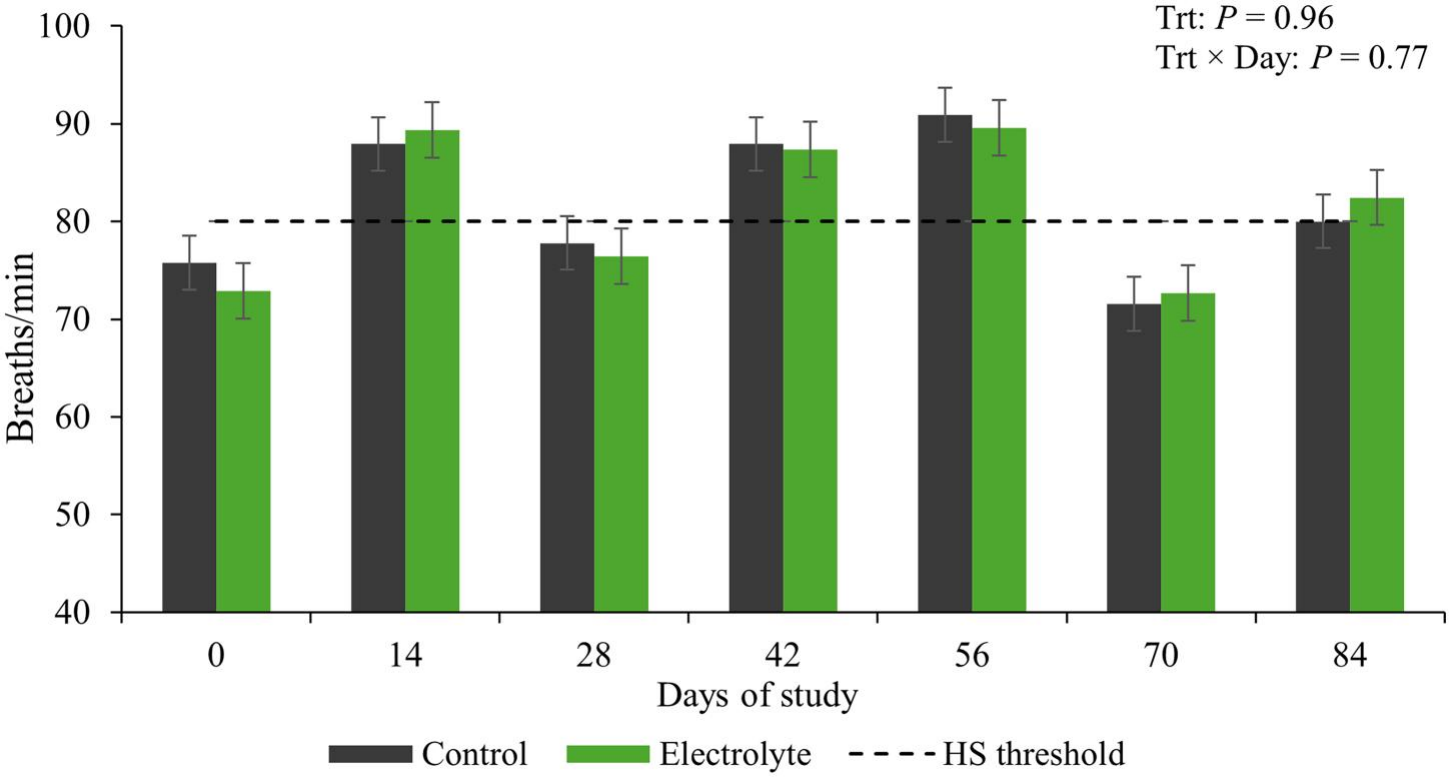
Respiration rate of beef cows supplemented or not supplemented with an electrolyte solution (5 pastures/treatment and 5 or 6 cows/pasture) from d 0 to 90 (90 ± 10 d pre-partum) every 14-days. Respiration rate was measured by visually counting flank movements of each cow for 1 min. Heat stress (HS) threshold (RR ≥ 80 bpm).

**Figure 5:**
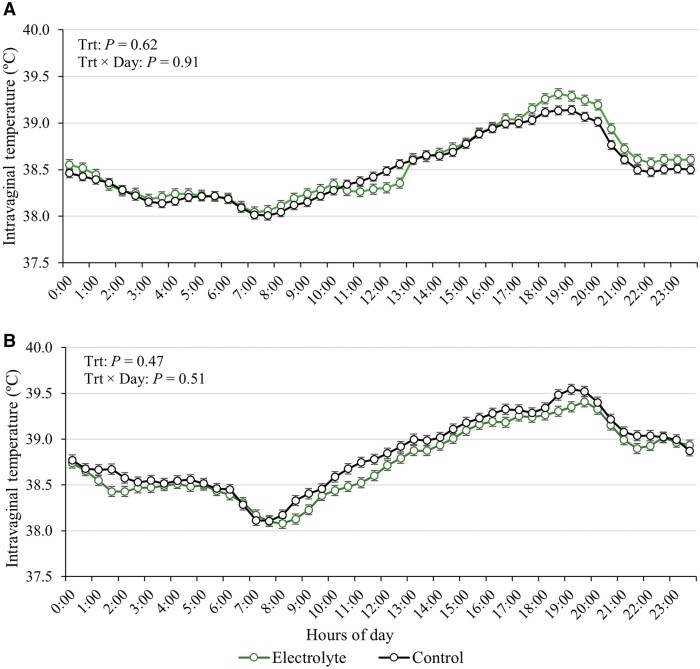
Intravaginal temperature (°C) from d 28 to 35 (Panel A) and d 56 to 63 (Panel B) of beef cows supplemented or not supplemented with an electrolyte solution (5 pastures/treatment and 5 or 6 cows/pasture) from d 0 to 90 (90 ± 10 d pre-partum). intravaginal temperature was averaged at 30-min intervals and then across days 27 to 34 (a), and days 55 to 62 (b) before statistical analysis. No effect of treatment or interaction between treatments was detected from d 28 to 35 (*P* ≥ 0.62) and from d 56 to 63 (*P* ≥ 0.47).

No effects of treatments or treatment × day interaction (*P* ≥ 0.77) were observed for RR (Fig. 4), and regardless of the treatment, cows exhibited RR over the threshold considered for moderate heat stress (RR ≥ 80 bpm) on d 14, 42, 56 and 84. No effect of treatment or treatment × day interaction was detected for intravaginal temperatures from d 28 to 35 (*P* ≥ 0.62; Fig. 5a) and from d 56 to 63 (*P* ≥ 0.47; Fig. 5b).

## Discussion

Most of the research on electrolyte supplementation for beef cattle has been focused on feedlot animals to alleviate the physiological disturbances associated with transportation stress, with supplementation primarily offered before transport and slaughter (Gortel et al. 1992; Schaefer et al. 1997; Beatty et al. 2007; Zhao et al. 2022). Exceptions include Ferreira et al. (2024), which examined electrolyte supplementation for preconditioning beef calves, and Smithyman et al. (2022), which studied newly received cattle. For dairy, electrolyte supplementation has been used as treatment for diarrhea in calves (Michell et al. 1992; Lorenz 2000; Constable et al. 2001), and to alleviate metabolic changes due to heat stress conditions in cows (Schneider et al. 1984; Mallonée et al. 1985; Wildman et al. 2007). Studies evaluating electrolyte supplementation for dairy cows under heat stress have reported variable outcomes, largely influenced by differences in experimental and climate conditions and animal category. In the realm of research focused on nutritional managements to mitigate heat stress, no studies have evaluated its effectiveness for beef cows in grazing systems in prolonged exposure to heat load.

In this study, cows were managed under prolonged exposure to high THI during gestation. Despite having access to shade, average RR indicated moderate to severe heat stress across treatments, as values exceeded the levels consistent with the upper limit of moderate heat stress (RR > 80 breaths/min; Brown-Brandl et al. 2005). The absence of differences in herbage mass, herbage allowance, and forage chemical composition between treatments suggests that the responses herein discussed were not influenced by forage availability or nutritive value, but rather by the treatments applied.

Lack of differences in performance observed in this study following electrolyte supplementation agrees with other published studies. Ferreira et al. (2024) evaluated electrolyte supplementation for preconditioning calves and did not find differences in growth performance. For dairy, electrolyte supplementation does not seem to change BW or BCS (Wildman et al. 2007; Cabrera 2014) although some have reported improved dry matter intake and milk yield (West et al. 1991; Sanchez et al. 1994). Differences in BW after electrolyte supplementation have only been reported in studies where electrolyte solution was offered before or after transportation. These studies have identified reduction of BW loss before (Schaefer et al. 1997) or during transportation (Beatty et al. 2007) following electrolyte supplementation and differences in hot carcass weight of bulls (Gortel et al. 1992).

The observed reduction in mineral intake for electrolyte-treated cows from wk 5 to wk 10 compared to control cows may be related to potential substitution effects, where Na consumption through electrolyte solution reduced the drive for mineral supplement intake. Electrolytes contain sodium and potassium, which are key components of mineral mixes, and this overlap could explain the decreased mineral consumption (McDowell 1992; Arthington and Ranches 2021). This might have contributed to lack of differences in blood Na between treatments.

The lack of treatment effects on respiration rate and intravaginal temperature indicates that electrolyte supplementation did not influence thermoregulation under the environmental conditions of this study. Beatty et al. (2007) observed a similar outcome during transportation, where heifers provided with electrolytes showed no differences in rectal temperature compared to controls, although treated animals tended to consume more water. In contrast, water intake in the present study was unaffected by the inclusion of electrolytes in drinking water, which positively suggests that the formulation did not alter water palatability.

In studies where electrolyte solutions and water are offered separately, total fluid intake may increase. For example, Ferreira et al. (2024) reported that electrolyte-treated calves consumed more total fluid, despite ingesting only about 20% of the electrolyte offered. Likewise, Smithyman et al. (2022) found that newly received heifers offered an electrolyte solution for three days post-arrival at the feedlot exhibited greater water intake compared to those provided only water.

Greater concentrations of ${\text{HCO}}_{3}^{-}$, BE, and tCO_2_, suggests a mild improvement in systemic buffering capacity. Similar trends have been reported where increased dietary cation-anion difference (DCAD) with electrolyte supplementation increased ${\text{HCO}}_{3}^{-}$ (West et al. 1991; Sanchez et al. 1994). Similarly, Beatty et al. (2007) observed that electrolyte supplementation in heifers during transportation contributed to maintaining acid–base homeostasis, as treated animals exhibited greater blood ${\text{HCO}}_{3}^{-}$ and BE concentrations compared to controls.

Specifically for animals under heat stress conditions, improving buffering capacity is critical to maintaining blood acid-base balance. Heat stress often leads to increased respiratory rate and metabolic alkalosis due to excessive CO_2_ loss (Khelil-Arfa et al. 2014). In respiratory alkalosis, the body tries to compensate by reducing ${\text{HCO}}_{3}^{-}$ via renal excretion (Schneider et al. 1988), when supplementation maintains or increases ${\text{HCO}}_{3}^{-}$ and BE, it prevents pH changes, stabilizing acid–base balance. Electrolyte supplementation for cows under heat stress builds on the rationale that it helps counteract shifts in acid-base balance for three main reasons: (1) potassium is the primary cation lost in bovine sweat (McEwan Jenkinson and Mabon 1973); (2) sodium is excreted along with bicarbonate to compensate for respiratory alkalosis that often occurs during heat stress (West 2003); and (3) the diurnal excretion of bicarbonates can induce metabolic acidosis during the cooler periods of the day (Schneider et al. 1988).

In this study, there was an improvement in the buffering capacity as mentioned above, but no changes in circulating Na and K were detected. Detectable differences in blood Na and K following electrolyte supplementation have been inconsistent between studies (Al-Qaisi et al. 2020; Ferreira et al. 2024; Cabrera 2014). These inconsistencies may be due to variations in the severity of the physiological insult (e.g., heat stress or transportation), differences in electrolyte formulation and intake (or DCAD of diet), animal demographics, and timing of sampling.

Cows receiving electrolyte supplementation exhibited lower hematocrit values, likely reflecting improved hydration status and plasma volume expansion. Under heat stress conditions, cattle experience increased water loss through sweating and respiration (West 2003), leading to dehydration and hemoconcentration (i.e., increased hematocrit due to reduced plasma volume). Electrolyte supplementation enhances water absorption and retention by promoting active transport of these ions in the gut, which drives water uptake through osmosis (Rao, 2019). This leads to an expansion of plasma volume, diluting the concentration of red blood cells in the blood, and therefore resulting in a lower hematocrit. This is also confirmed by lowered albumin concentrations for electrolyte cows on days 56 and 84. Albumin is the primary blood protein, and amongst other functions, binds metabolites and regulates plasma osmolality (Quinlan et al. 2005). During dehydration, for example, plasma volume decreases, causing albumin concentration to rise. Previous studies have documented similar reductions in albumin following fluid therapy in cattle (Smith 2005). Therefore, together, lower hematocrit and albumin for electrolyte supplemented cows may reflect hemodilution associated with improved hydration status (Constable et al. 2001). For bulls receiving water or electrolytes prior to slaughter, no differences were observed in glucose, beta-hydroxide butyrate, hematocrit, or plasma electrolytes (Gortel et al. 1992; Schaefer et al. 1992).

In this study, electrolyte supplementation was mixed directly into water tanks, and supplemented cows did not have free choice access to plain water. Future research should explore strategic, short-term electrolyte supplementation during the most challenging heat stress periods rather than prolonged administration. This approach may help minimize undesirable effects on loose mineral intake. Additionally, conducting preference trials could be considered to identify the most effective and practical methods for delivering electrolytes under heat stress conditions, ensuring both palatability and adequate intake.

## Conclusions

Electrolyte supplementation for black-hided beef cow subjected to prolonged heat stress conditions improved hydration status and acid-base balance, suggested by reduced hematocrit and albumin, and increased bicarbonate and base excess. However, this did not translate into improvements in performance or thermotolerance. Electrolyte supplementation did not affect water intake, but reduced mineral intake, which might have offset the benefits of electrolyte supplementation.

## Supplementary Material

txag031_Supplementary_Data

## List of Abbreviations

BCS: Body condition score
BE: Base excess
CP: Crude protein
DM: Dry matter
HCO₃⁻: Bicarbonate
iCa: Ionized calcium
**K** ^**+**^: Potassium
pCO₂: Partial pressure of carbon dioxide
pO₂: Partial pressure of oxygen
RR: Respiration rate
**Na** ^**+**^: Sodium
NDF: Neutral detergent
NEg: Net energy for gain
NEm: Net energy for maintenance
**SO**_**2**_: Oxygen saturation
SUN: Serum urea nitrogen
tCO₂: Total carbon dioxide
TDN: Total digestible nutrients
THI: Temperature-humidity index
TDN: Total digestible nutrients
