# Supplementary material for: Electrolyte supplementation for gestating beef cows under heat stress conditions: cow-calf performance, thermotolerance and physiological responses
Source: Transl Anim Sci. 2026 Mar 16;10:txag031. doi: 10.1093/tas/txag031 (PMC13049185; doi:10.1093/tas/txag031)
Supplement: txag031_Supplementary_Data [file txag031_supplementary_data.docx]

**Supplementary file 1**. Predicted (x axis) and Observed (y axis) of water recovery estimated with a turbine flow meter. Accuracy testing involved 20 measurements in which water was removed from the tank, its volume manually measured and then compared to the corresponding readings from the flow meter.
